# Spatiotemporal change characteristics of vegetation coverage in Shangwan Mine of China’s Shendong Mining Area

**DOI:** 10.1371/journal.pone.0302278

**Published:** 2024-04-29

**Authors:** Ziheng Song, Jie Fang, Jian Zhang, Gang Liu, Liping Sun, Chuangang Gong, Fei Wang

**Affiliations:** 1 State Key Laboratory of Water Resources Protection and Utilization in Coal Mining, Beijing, China; 2 School of Mining and Geomatics Engineering, Hebei University of Engineering, Handan, China; 3 Shenhua Shendong Coal Group Company Limited, Ordos, China; 4 Nanchang Institute of Technology, Nanchang, China; 5 National Institute of Energy Economics and Technology Company Limited, Beijing, China; 6 School of Geomatics, Anhui University of Science and Technology, Huainan, China; Van Lang University: Truong Dai hoc Van Lang, VIET NAM

## Abstract

The coal mining might cause the disturbance to the vegetation and the disturbance impacts might exist the differences for different areas, and few literatures compared and analyzed different disturbed areas based on the location of the mining face, and paid attention to the post mining self-healing effects of vegetation. Here, this paper selected the GaoFen multispectral images during 2017–2021 to study different areas of Shangwan Mine which includes the old mining area more than 5 years after mining, the new working face underground mined in 2018 and 2019, the natural growth control area and the open-pit mining affected area. The spatiotemporal changes of the surface fraction vegetation coverage (*FVC*) were analyzed in each area and the correlation between vegetation coverage and climatic factors was studied. The results showed that: (1) The overall vegetation coverage showed a moderate decrease trend in fluctuation from 2017 to 2021. The Open-pit mining affected areas showed the largest decline, reaching 68.3%. The *FVC* in the underground mining areas had a downward trend, but self-healing effect after mining was also observed. (2) The overall *FVC* in the study area was positively correlated with the number of precipitation days. (3) There were differences in the sensitivity to mining disturbance for different landform in the underground mining areas. (4) Although the *FVC* in the Old mining areas had recovered to the level of Natural growth control area, but the annual fluctuation was larger, which might mean lower ecological stability.

## Introduction

Shendong Mining Area is an important coal mining base in China, providing strong support for China’s economic development. In recent years, countries around the world have gradually reached a consensus on environmental protection. Under the concept of sustainable development, China has put forward the development concept of "green water and green mountains are invaluable assets" and other development concepts that coordinate between energy mining and ecological protection [1]. Scientifically grasping the impact of coal mining on the surface ecological environment can provide a scientific basis for ecological governance and pre-mining ecological disturbance assessment. Coal mining may cause land occupation, surface subsidence, disturbance of soil structure, damage to vegetation roots, accelerate soil moisture loss, and have a certain impact on the ecological environment of surface vegetation, especially vegetation coverage [2]. The impact of coal mining on surface vegetation coverage is complex and it is difficult to determine whether there is a self-healing effect after mining. Self-healing refers to the self-healing trend exhibited by plants or communities on the surface vegetation under natural growth conditions without human intervention after being affected by environmental disturbances. Current studies have found that mining subsidence has a certain negative effect on surface vegetation which includes root damage and changes of soil moisture and nutrient content [3, 4]. However, there are also studies suggesting that vegetation may have a self-healing effect after disturbance. The self-healing ability of plants is an important foundation for the self-healing ability of vegetation communities [5, 6]. Therefore, disturbances caused by mining usually need to be tracked and monitored continuously in order to obtain the disturbance law.

Currently commonly used monitoring methods for coal mining induced vegetation disturbance can be divided into two categories: (1) Field sampling methods. Plots are set to analyze the *FVC* spatiotemporal changes and species diversity in the mining area [7–9]. However, due to the complex landform changes, it is necessary to lay out enough density of the sample squares to reflect the change law of the entire subsidence area which would be labor-intensive and time-consuming. (2) Remote sensing monitoring methods. At present, there are a large number of researches to monitor the disturbance in mining areas based on satellite images due to the ability of rapid observations in large areas [10–16]. Vegetation cover was one of effective indicators to detect the disturbance and the dynamics could reflect the vegetation damage, land reclamation and vegetation recovery [13–16]. For example, Yang et al. [14] detected and mapped the vegetation disturbance and recovery for each year according to the Normalized Difference Vegetation Index(*NDVI*) of Landsat. Yu et al. [13] studied spatiotemporal variation of vegetation cover of mining areas from 2005 to 2020 in Dexing city and found that it was stable possibly due to actively planting vegetation and land reclamation. Zhang et al. [16] used *FVC* to analyze the vegetation change and restoration degree in the mining area, especially that around the dumps with Landsat data. The disturbance impacts on the vegetation may exist the differences for different areas such as the coal mining area and nature growth area. However, most of studies focused on the disturbance impacts in the entire mining area, and few literatures compared and analyzed different disturbed areas based on the location of the mining face, and paid attention to the post mining self-healing effects of vegetation.

To explore the impact of different mining disturbance on vegetation coverage and post mining self-healing of vegetation, this paper focused on working face and selected the GaoFen multispectral images to study different areas of Shangwan Mine which includes the old mining area more than 5 years after mining, the new working face underground mined in 2018 and 2019, the natural growth control area, and the open-pit mining affected area. Thus, the objectives of this study were (1) to analyze and reveal spatiotemporal variation characteristics of vegetation cover in the entire mining area and in each subdivided area, (2) to explore the relationship between vegetation cover and climate factors through correlation analysis, and (3) to analyze the self-healing law of vegetation after underground mining disturbance.

## Materials and methods

### Study area

**Fig 1:**
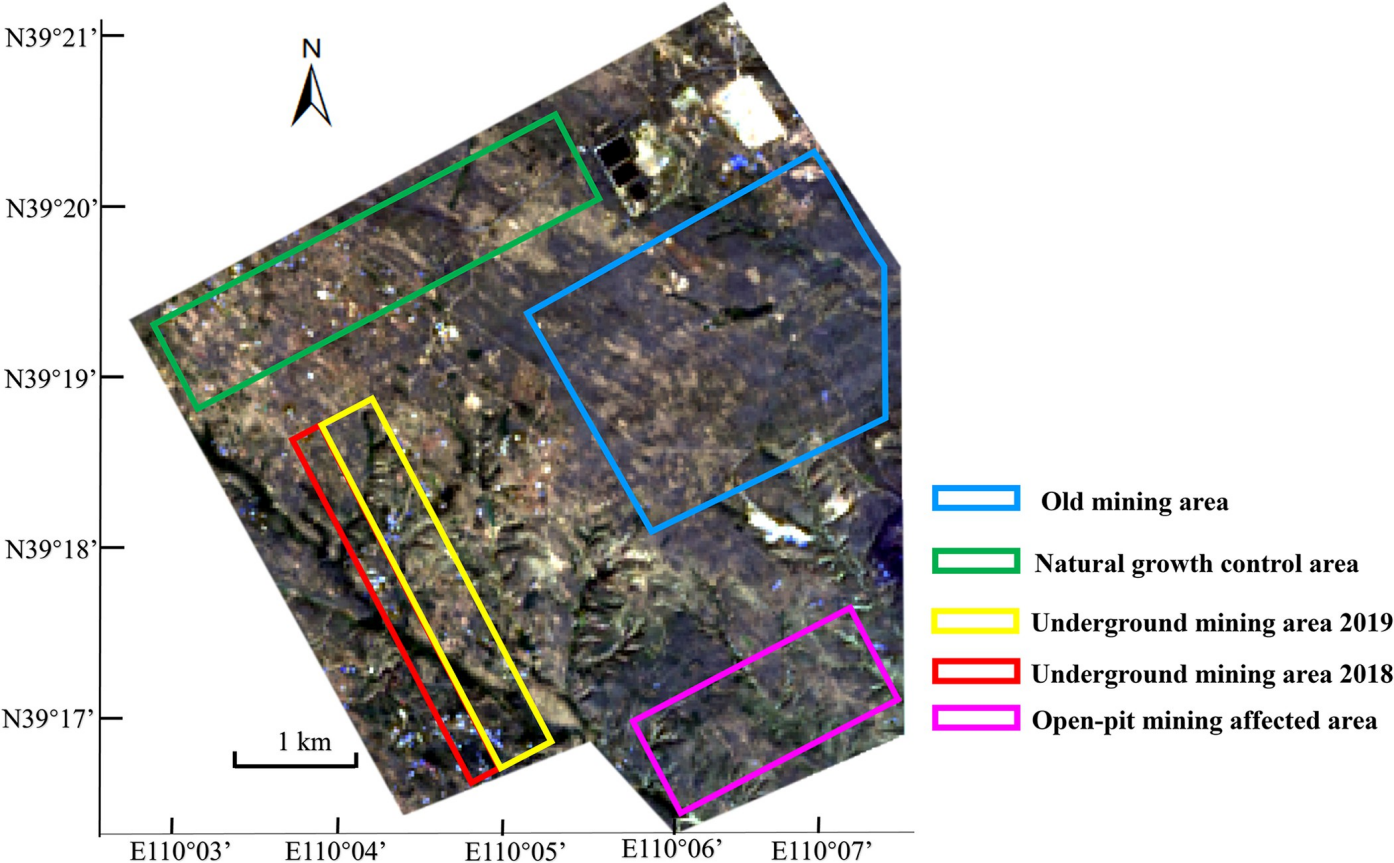
Analysis area distribution in the study area. This figure is only for illustrate the location of the research area and the distribution of each analysis area. The base map is image from Landsat (https://landsat.visibleearth.nasa.gov/).

The study area of Shangwan Mine is located in the intersection of the Loess Plateau in northern Shaanxi and the Mu Us Sandy Land in Inner Mongolia (E110°02’53"-E110°08’24", N39°16’20"-N39°20’58") as shown in Fig 1. The study area has a temperate plateau climate. Soil erosion was serious. The local plants are mainly shrubs and herbs. The dominant shrubs are arternisia ordosica and caragana microphylla and the dominant herbs are stipa glareosa and leymus. The local soil is mainly sandy soil and sandy loam while clay loam is with small distribution, and the soil is weakly alkaline. Over 60% of the total annual precipitation, which averages 437.5 mm, falls during the months of July-September. The region has an average elevation of 1200 m, with an annual average sunshine of 2876 hours and an average temperature of 8.4°C. The landforms are characterized by a fragmented surface, with complex fractured features such as large gullies on the southern side, while the northern side is relatively flat.

To explore the small-scale ecological effect focusing on working face, this paper divided the study area into different disturbance areas according to the design map of underground mine as shown in Fig 1. The disturbance areas included ① Old mining area, located on the northeast side of the study area. During the period from 2002 to 2009, underground mining subsidence occurred, and reclamation was carried out with planted trees (Pinus sylvestris, Siberian apricot) and shrubs (Artemisia annua, Caragana). After reclamation was completed, it was allowed to grow naturally. ② Natural growth control area, located on the northwest side. The landform was relatively flat, and the vegetation in this area was mostly herbs and shrubs. ③ Underground mining area 2019, with strip shape. It was with flat landform on the north side and fragmented landform on the south side. The working face in this area was mined in 2019, with 600m of mining width. ④ Underground mining area 2018. The landform was similar to the area ③ and the mining width was 300 m. ⑤ Open-pit mining affected area, located on the northern of an open-pit mine. It was found that the surface was greatly affected by the open-pit mine through investigation. The open-pit mine was put into use in the early stage and the technical transformation was completed in 2007 with an output of about 3 million tons/a.

### Experimental data

The used multispectral images in this study were captured by the satellite of the GF-1 and GF-6 as shown in Table 1. To minimize the impacts of phenological changes, the same month images on August of each year were selected for the study since they were flourishing at this time. Images during 2017–2020 were obtained from the satellite GF-1 while the remaining image was captured by the satellite GF-6. Two single images with overlapping areas were merged into the image of 2017 with the ENVI software since one single image could not completely include the study area. The spatial resolution of used multispectral images were 8 m and the cloud cover was less than 5%. To verify the reliability of the results, this paper downloaded Lansat8 OLI-TIRS data from 2017 to 2021as shown in Table 2 and calculated the *FVC* of each year, and then made a comparative analysis between the GaoFen data and Landsat data. The meteorological data used was from the China Meteorological Data Center(http://data.cma.cn).

**Table 1:** Information of GaoFen data selected.

Years	Satellite	Date	Satellite sensor
2017	GF-1	2017-08-11	PMS1+2
2018	GF-1	2018-08-15	PMS1
2019	GF-1	2019-08-07	PMS2
2020	GF-1	2020-08-10	PMS2
2021	GF-6	2021-08-16	PMS

**Table 2:** Information of Landsat data for validation.

Years	Satellite	Date	Satellite sensor
2017	Landsat 8	2017-09-08	OLI_TIRS
2018	Landsat 8	2018-07-25	OLI_TIRS
2019	Landsat 8	2019-08-29	OLI_TIRS
2020	Landsat 8	2020-09-16	OLI_TIRS
2021	Landsat 8	2021-08-02	OLI_TIRS

### Data processing and operation

#### Image preprocessing

The image was first orthorectified using the RPC orthorectification tool with software ENVI 5.3. In order to ensure that the images in each period achieve pixel-level alignment, the Image Registration Workflow module was used to register the images in pairs. The data of 2018 was regarded as the base image and the matching model used Cross Correlation in which the control point accuracy was required to be set below 1.0.

Then radiance calibration and atmospheric correction were performed. Radiance calibration was carried out with the conversion relationship between the observed value and the radiance value as shown in Formula (1) [17]:

$$L_{\lambda }=Gain\times DN+Bias$$ (1)

Where *L*_*λ*_ represents the spectral radiance at the entrance pupil of the sensor, *DN* is the digital number, *Gain* is the scaling gain.

Atmospheric correction was performed in the FLAASH module, and vegetation spectrum before and after correction were compared to verify the accuracy of the correction results.

#### Calculation of vegetation coverage

*NDVI* was one of the most commonly used spectral vegetation indexes to reflect the distribution of surface vegetation and it was calculated with the reflectance of near-infrared band and red band as shown in Formula (2) [18].


$$\mathrm{NDVI}=\frac{\rho_{\mathrm{NIR}}‐\rho_{\mathrm{RED}}}{\rho_{\mathrm{NIR}}+\rho_{\mathrm{RED}}}$$ (2)

Where *ρ*_*NIR*_ represents the reflectance of the near-infrared band, and *ρ*_*RED*_ is the reflectance of the red band.

Previous studies have shown that *NDVI* was linearly correlated with vegetation coverage, and could be used to estimate *FVC* [19]. This study used the dimidiate pixel model to calculate *FVC* in which the pixel was supposed to be only composed of vegetation and non-vegetation as shown in Formula (3).


$$\mathrm{FVC}=\frac{\mathrm{NDVI}‐{\mathrm{NDVI}}_{\mathrm{soil}}}{{\mathrm{NDVI}}_{\mathrm{veg}}‐{\mathrm{NDVI}}_{\mathrm{soil}}}$$ (3)

Where *NDVI* represents the *NDVI* value of the desired pixel; *NDVI*_*soil*_ represents the *NDVI* value of a pixel without vegetation coverage; while *NDVI*_*veg*_ stands for the *NDVI* value of a pixel with complete vegetation coverage. Theoretically, *NDVI*_*soil*_ and *NDVI*_*veg*_ should be close to 0 and 1, respectively, but the values would change with time and regions and were not fixed in fact. *NDVI*_*soil*_ and *NDVI*_*veg*_ were always determined by the confidence level and respectively corresponded to the value at lower and upper 5% based on the cumulative distribution of *NDVI* [13].

In order to reflect the spatial distribution of the *FVC*, it could be classified into the following five grades as shown in Table 3 according to classification method of vegetation coverage grades [18].

**Table 3:** Classification table of vegetation coverage.

*FVC*	Grade	Level	Description
0≤*FVC*≤0.2	Ⅰ	Low coverage	Bare ground, sand
0.2<*FVC*≤0.4	Ⅱ	Low to medium coverage	Less vegetation
0.4<*FVC*≤0.6	Ⅲ	Moderate coverage	Moderate cover, sparse shrubs
0.6<*FVC*≤0.8	Ⅳ	Medium to high coverage	Moderate cover, low shrubs
0.8<*FVC*≤1	Ⅴ	High coverage	High cover shrubs, trees

#### Time series trend analysis of vegetation coverage

A univariate linear regression model [20] was used to fit the *FVC* of each pixel from 2017 to 2021, and the slope value (*θ*_*slope*_) of the resulting trend line was calculated for each pixel. The building areas were not involved in the calculation. When the slope value is negative, it indicates a degradation trend in vegetation, and the magnitude of the slope value represents the degree of degradation, with larger absolute values indicating more severe degradation. On the contrary, positive value represents increasing trend of vegetation coverage, and the larger the absolute value, the more obvious the increasing trend. The change trend significance was evaluated using the correlation coefficient (*R*). The calculation methods of *θ*_*slope*_ and *R* are as shown in Formula (4) and (5) [18].


$$\theta_{\mathrm{slope}}=\frac{\sum_{i=1}^{n}x_{i}t_{i}‐\frac{1}{n}(\sum_{i=1}^{n}x_{i})(\sum_{i=1}^{n}t_{i})}{\sum_{i=1}^{n}t_{i}^{2}‐{\frac{1}{n}(\sum_{i=1}^{n}t_{i})}^{2}}$$ (4)

$$R=\sqrt{\frac{\Sigma_{i=1}^{n}t_{i}^{2}‐{\frac{1}{n}(\Sigma_{i=1}^{n}t_{i})}^{2}}{\Sigma_{i=1}^{n}x_{i}^{2}‐{\frac{1}{n}(\Sigma_{i=1}^{n}x_{i})}^{2}}}\theta_{\mathrm{slope}}$$ (5)

Where *t*_*i*_ is the year order, *x*_*i*_ is the *FVC* of the *i* th year.

*T*-test was selected to verify whether exists the real relevance between two variables and the significant level of the variation trend. Vegetation coverage change trend could be classified into the following five degrades according to *θ*_*slope*,_ *R* and significance as shown in Table 4.

**Table 4:** Vegetation coverage growth trend grading.

Grade	*θ*_*slope*_	*P*	*R*
Significant decrease	<0	*P*<0.05	-1≤*R*<-0.58
Tended decrease	<0	0.05≤*P*	-0.58≤*R*≤0
Significant increase	>0	*P*<0.05	0.58<*R*≤1
Tended increase	>0	0.05≤*P*	0≤*R*≤0.58
Stable	0	\	\

#### Annual change of vegetation coverage

To study the annual change of *FVC* in the divided areas, the vegetation coverage change rate was defined as shown in Formula (6):

$$V=(FVC_{n}‐FVC_{n‐1})/FVC_{n‐1}$$ (6)

Among them, *V* is the vegetation coverage change rate, *n* is the year, and *FVC*_*n*_ is the vegetation coverage in *n* years.

#### The variation characteristics of *FVC* at different distances from the working face after mining

**Fig 2:**
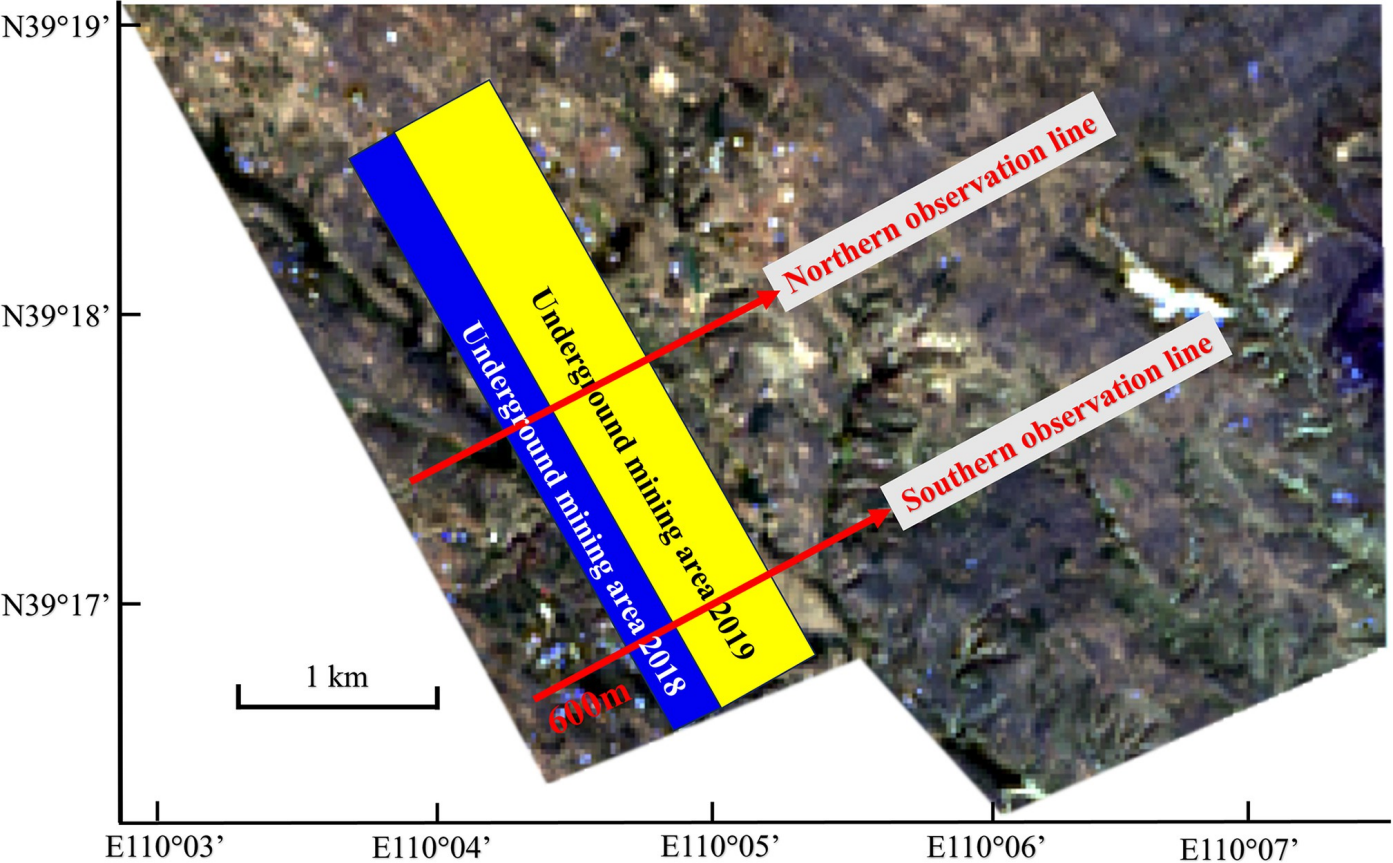
Location of the observation lines. This figure is only for illustrate the location of the research area and the location of the observation lines. The base map is image from Landsat (https://landsat.visibleearth.nasa.gov/).

Generally speaking, the disturbance and self-healing effects are difficult to detect only comparing the *FVC* on working face and natural growth areas in the mining year since there exist the vegetation coverage differences in the initial state due to the geomorphological factors. Thus, to further analyze the change laws in different regions before, during and after mining, two observation lines were set through the working face. One observation line was selected for the fragmented landform area on the south side and the other was selected for the flat area on the north side as shown in Fig 2. The change trend curve was generated in ENVI 5.3.

## Results and analysis

### Temporal and spatial distribution of vegetation coverage in the entire study area

**Fig 3:**
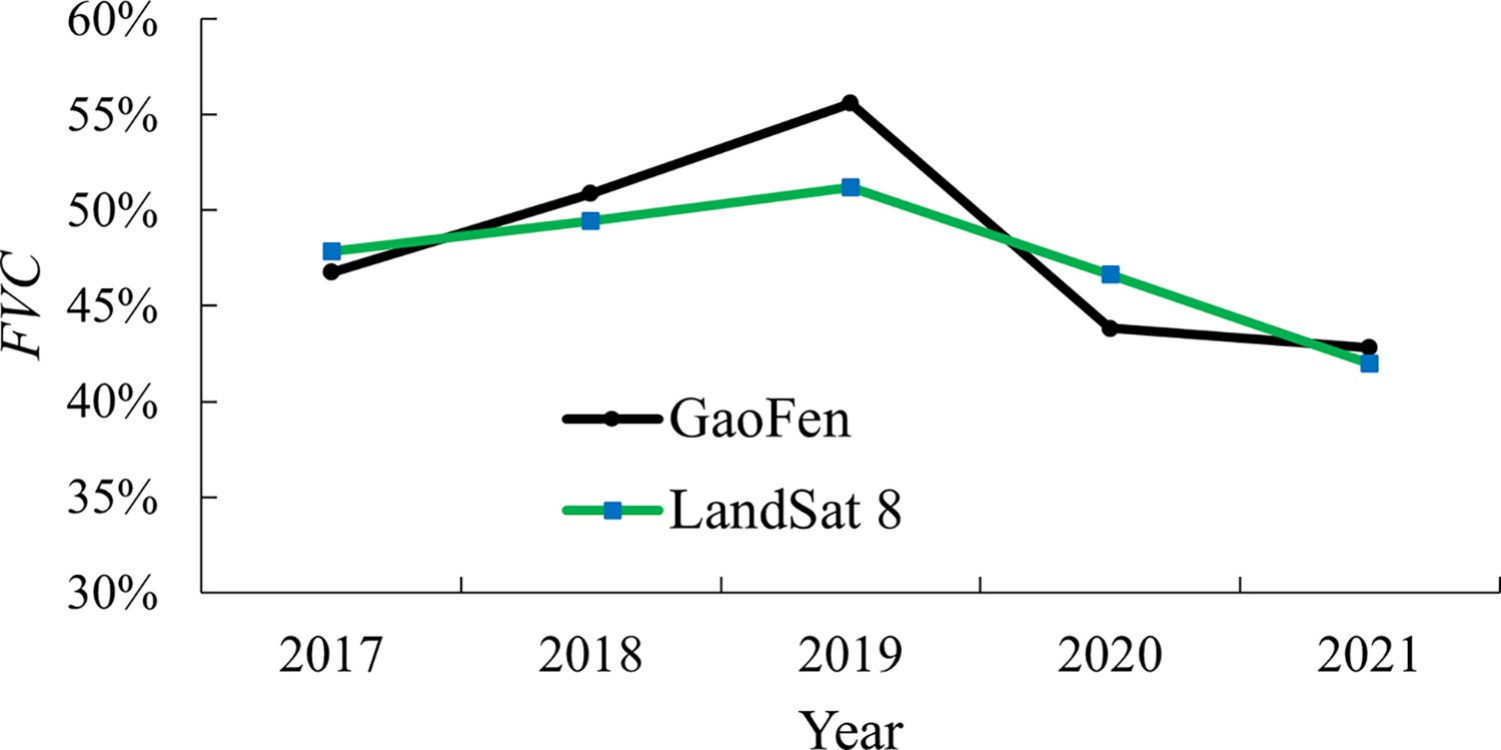
*FVC* variation based on Landsat and GaoFen satellites.

The *FVC* of the entire area during 2017–2021 was computed as shown in Fig 3, which showed that it increased during 2017–2019, but it demonstrated a downward trend during 2020–2021. To verify the result, the experiment was also carried out based on the Landsat data. The results in Fig 3 showed that both *FVC* have the same trend in changes, and the differences between them were small for each year and the largest one was within 10%, which further improved the reliability of our results.

**Fig 4:**
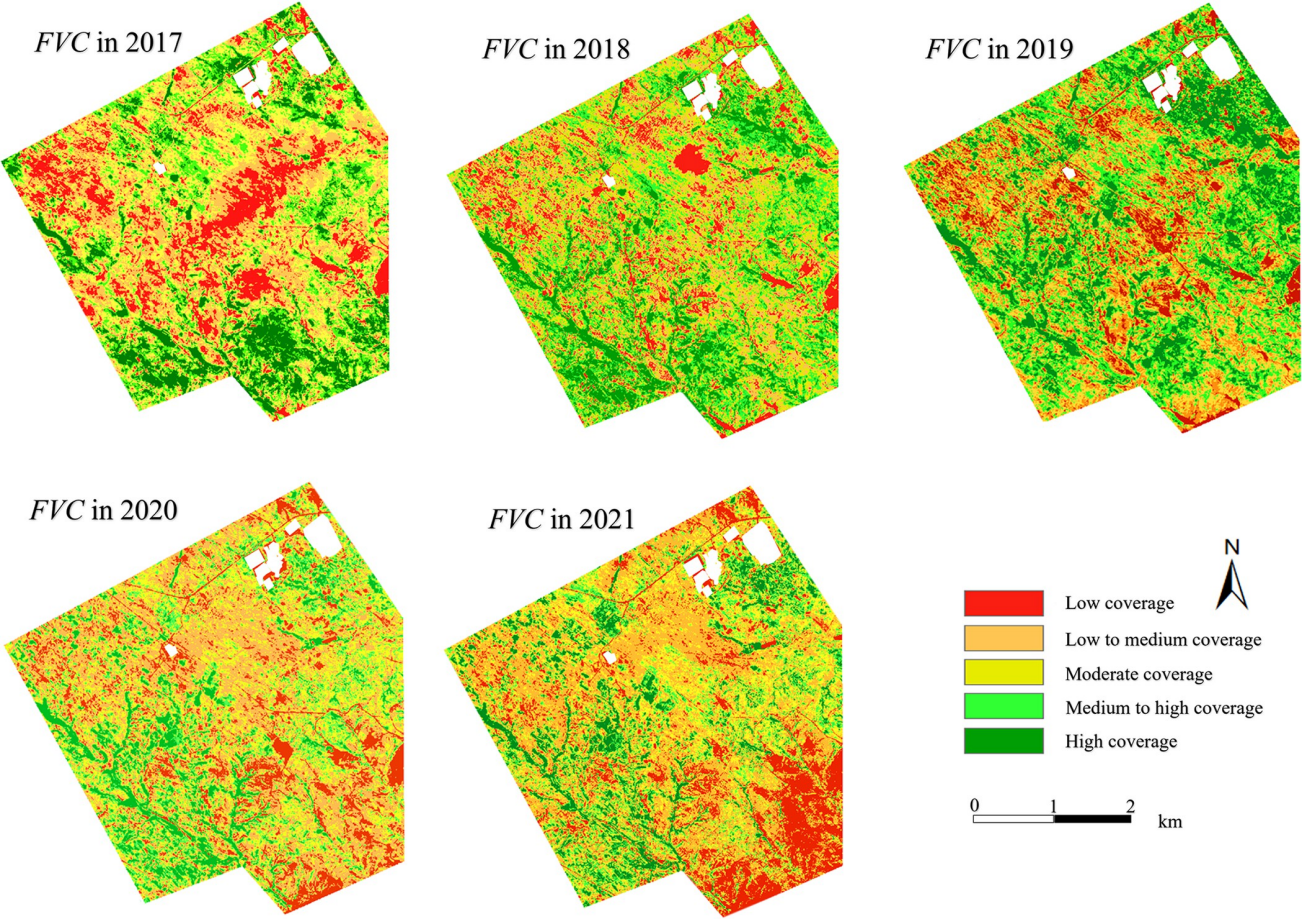
Temporal and spatial distribution of vegetation coverage in the study area. This figure were generated with ESRI using author-owned GaoFen data.

**Fig 5:**
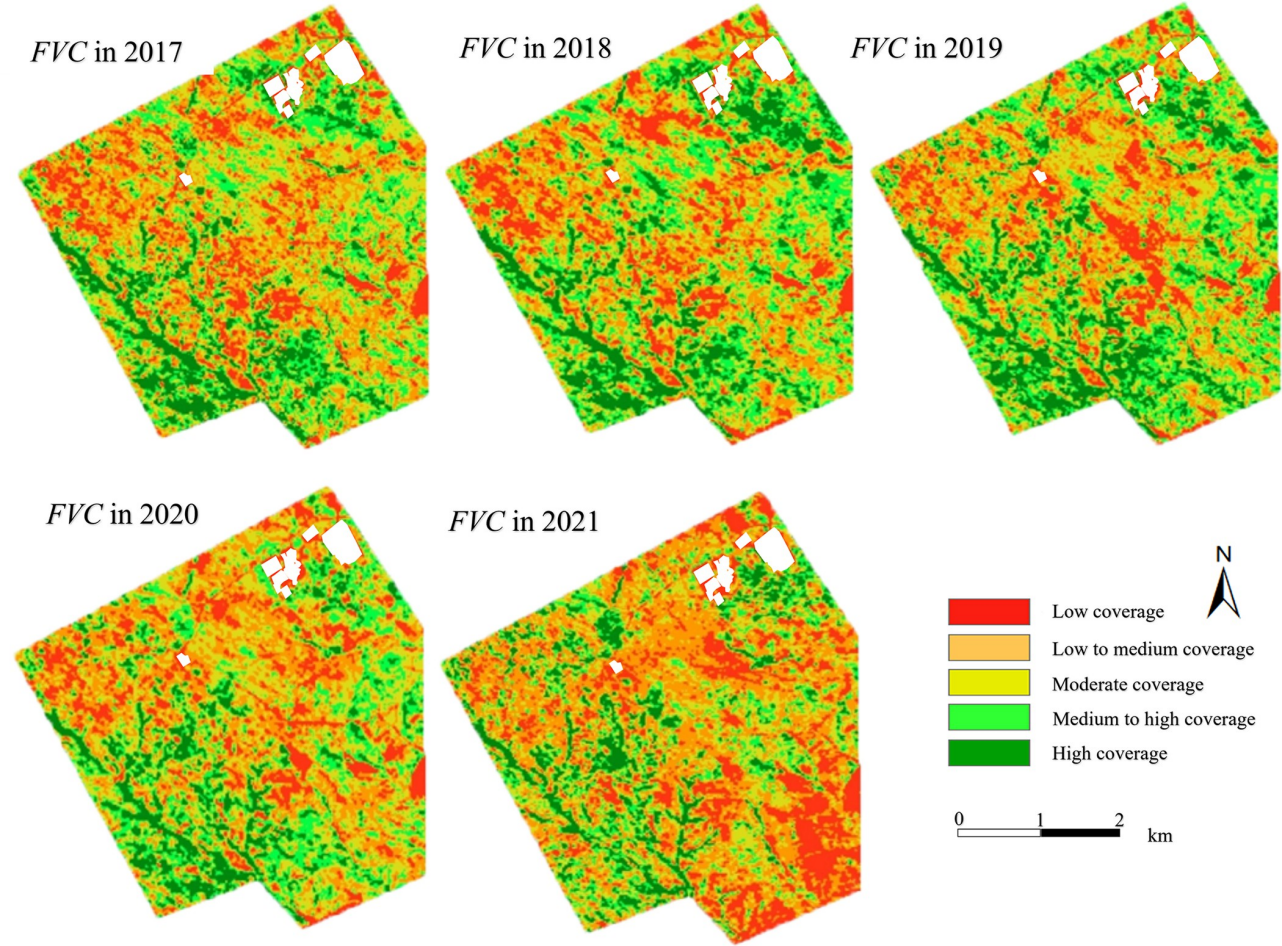
Temporal and spatial distribution of vegetation coverage in the study area. This figure were generated with ESRI using author-owned Landsat data.

The spatiotemporal distribution map of *FVC* was generated based on GaoFen data as shown in Fig 4. Overall, vegetation coverage in 2017 was relatively low, and most of them was below the medium-low coverage. The *FVC* was relatively higher in the fragmented landform area on the south side. In subsequent years, the *FVC* has an increasing trend, and reached the peak of *FVC* in 2019. However, the *FVC* in the fragmented area on the south side did not increase significantly, and even showed a downward trend. Then the overall *FVC* showed a decrease trend during 2020–2021, especially in the Open-pit mining affected area on the south side, there was a large area with low-level vegetation coverage. We also performed the experiment based on the Landsat data, which showed both of the spatiotemporal distribution were basically consistent as shown in Fig 5. This further proved the reliability of the research results. Unless otherwise specified, the mentioned *FVC* in the subsequent texts are all from GaoFen data.

**Fig 6:**
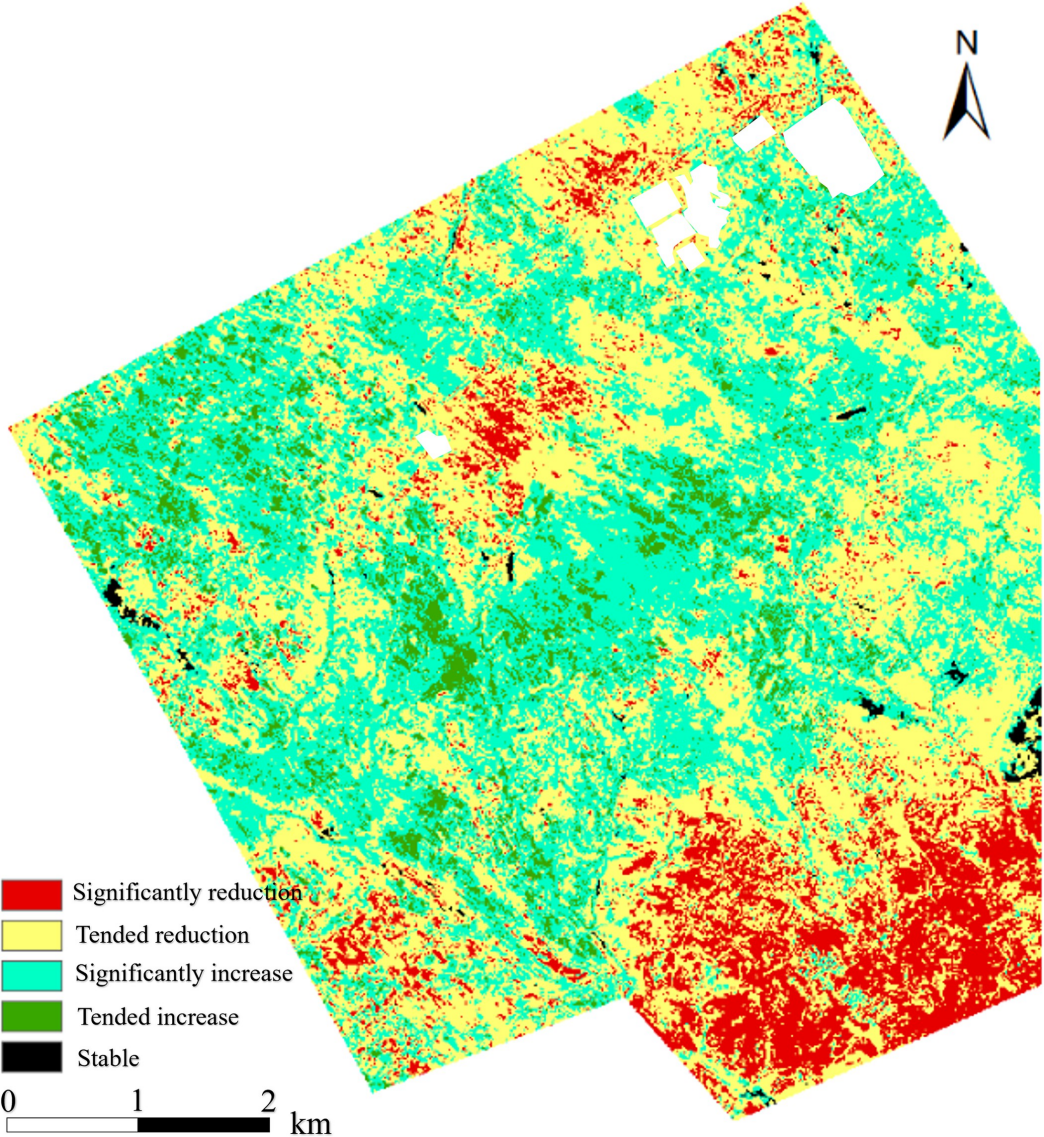
Univariate linear fitting of vegetation coverage time series change rate. This figure were generated with ESRI using author-owned data.

Change trend of vegetation coverage during 2017–2021 was obtained according to the univariate linear regression model as shown in Fig 6. The vegetation coverage on the north side was relatively stable, but that on the south side of the entire region was degraded, especially for the Open-pit mining affected area, significant vegetation degradation occurred.

### Time series variation characteristics of vegetation coverage in each analysis area

**Fig 7:**
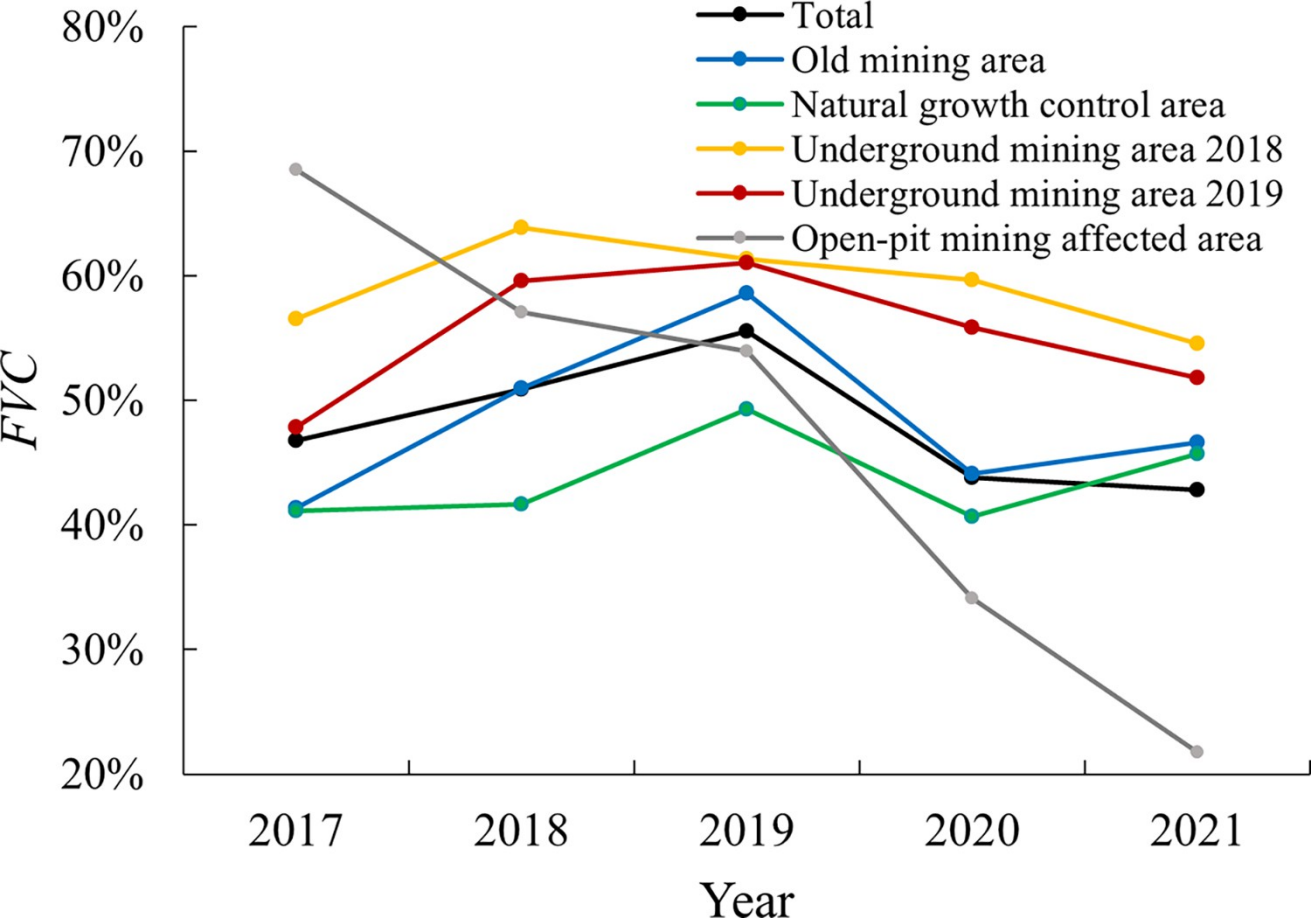
Time series change of *FVC* in each analysis area.

The *FVC* in each analysis area was shown in Fig 7. The *FVC* generally showed a trend with an upward during 2017–2019, a decline in 2020, and a further increase in 2021. Among them, the *FVC* in 2021 was 8.5% lower than that in 2017. Although the time-series change laws of the Natural growth control area and the Old mining area were consistent with that of the entire area, the fluctuation of the Old mining area was higher. For the Underground mining area 2018, the trend was opposite to that of the mining area which showed a downward trend in 2018 and 2019, and also continued to be slight downward in the subsequent years. The same pattern also appeared for the Underground mining area 2019 showing a continuous downward trend since the year of mining. This indicated that the coal mining would negatively affect the vegetation. Compared with other research areas in the same period, the *FVC* in the Open-pit mining affected area declined continuously.

According to the change trend of vegetation coverage as shown in Fig 6, the *FVC* in the Natural growth control area mainly showed a significant increase. The vegetation growth and degradation parts of the Old mining area were equivalent, and there was no significant change observed. For the Underground mining area 2018 and area 2019, the *FVC* of the fragmented landform on the south side was significantly reduced, and the north side was relatively stable.

**Fig 8:**
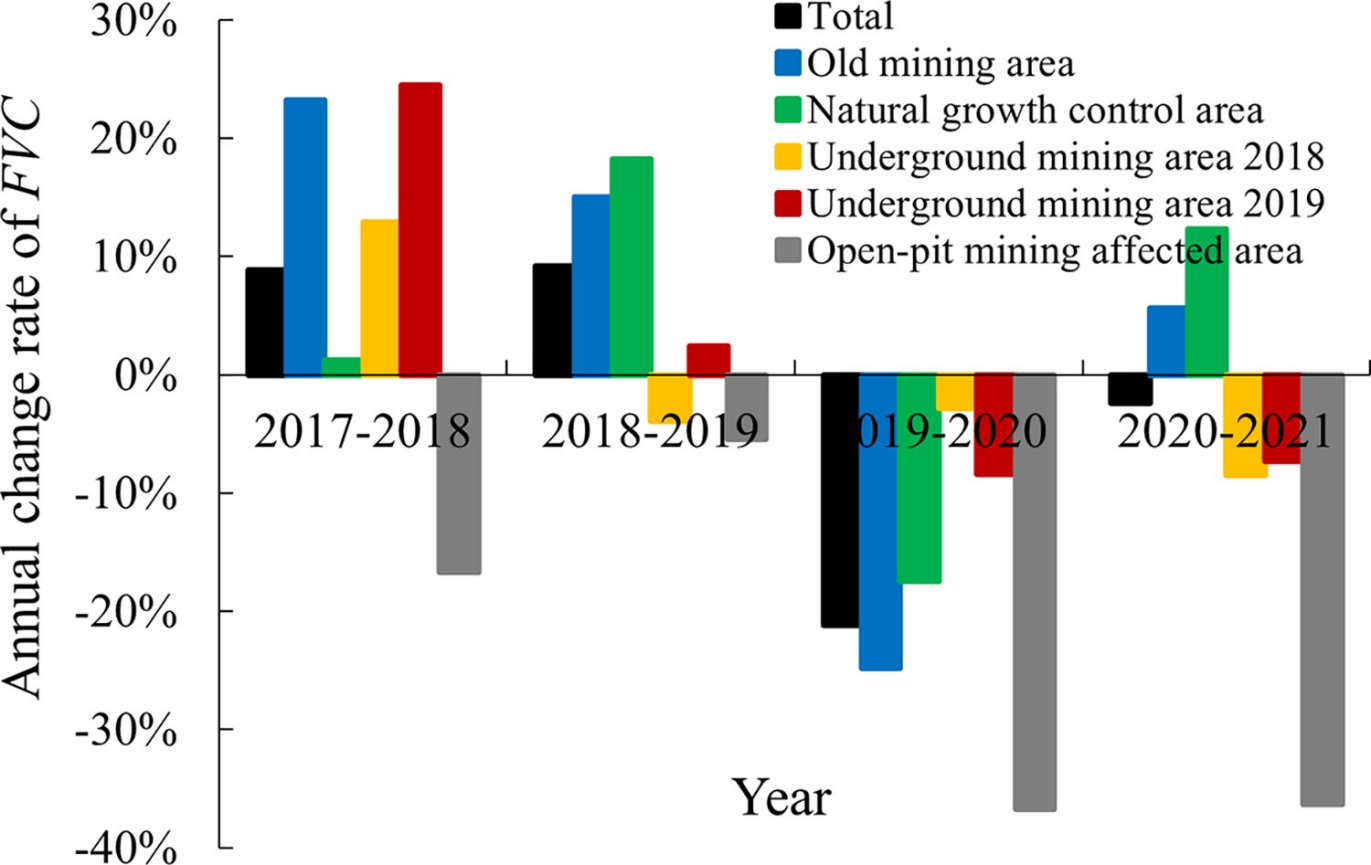
Annual change rate of *FVC* in each analysis area.

The annual change rate of *FVC* was obtained as shown in Fig 8. The Underground mining area 2018 and area 2019 were geographically adjacent, but in 2017–2018, *FVC* growth rate of the Underground mining area 2019 was 89.6% higher than that of Underground mining area in 2018. However, both areas were disturbed in 2018–2019, and the growth rates of both were significantly reduced. This disturbance continued to be observed in subsequent years.

### Correlation analysis of *FVC* and climate parameters in each analysis area

To reveal the relationship between vegetation cover and climate factors, the correlation analysis between the mean *FVC* and the climate parameters (precipitation, precipitation days, and average temperature data from March 1st in the vegetation growth season to the end of the study month on September 1st) was carried out as shown in Table 5. The correlation between *FVC* and precipitation for each analysis area was relatively low, while the correlation with precipitation days was relatively high, and they were all positive correlations above the medium correlation level. The correlation coefficients of the mining-affected areas were higher than that of the Natural growth control area and they were all strongly positively correlated with the number of precipitation days (0.6<r<0.8), while the natural growth control area was moderately positively correlated (0.4<r<0.6).

**Table 5:** Correlation analysis between vegetation coverage and climate in each analysis area.

		Pt	Ptdays	Tmp	*FVC*					
					Total	OldM	Control	WellM18	WellM19	OpenM
Pt		1.000								
Ptd		-0.434	1.000							
Tmp		-0.190	0.144	1.000						
*FVC*	Total	-0.477	0.982	0.152	1.000					
	OldM	-0.074	0.776	0.014	0.846	1.000				
	Control	-0.120	0.405	-0.421	0.534	0.791	1.000			
	WellM18	0.015	0.772	0.517	0.706	0.588	-0.025	1.000		
	WellM19	0.270	0.707	0.199	0.700	0.853	0.413	0.844	1.000	
	OpenM	-0.829	0.659	0.323	0.604	0.090	-0.170	0.418	0.009	1.000

Pt: precipitation; Ptd: precipitation days; Tmp: temperature; OldM: Old mining area; Control: Natural growth control area; WellM18: Underground mining area 2018; WellM19: Underground mining area 2019; OpenM: Open-pit mining affected area.

### Vegetation distribution changes of the observation line through working face

**Fig 9:**
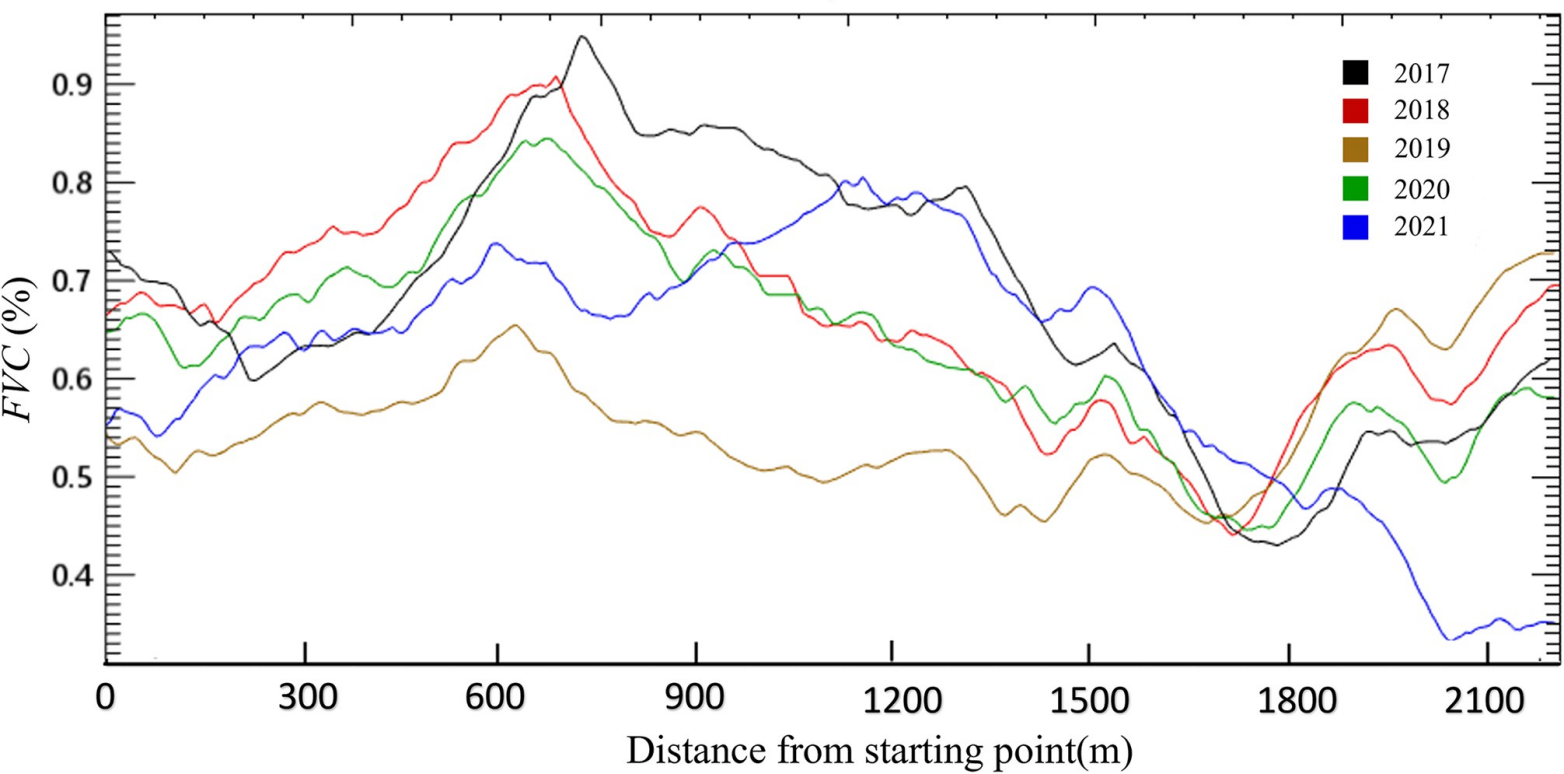
*FVC* change of southern observation line.

The change trend of *FVC* was obtained for the southern observation line that crossed through two working faces underground mined in 2018 and 2019 as shown in Fig 9. Area underground mined in 2018 was located at 600–900 m on the horizontal axis while area underground mined in 2019 was located at 900–1500 m on the horizontal axis. The landform in the southern observation line was relatively fragmented. It could be observed that mining had a negative impact on vegetation coverage, but there was also a significant self-healing effect after mining. The vegetation coverage on the working faces in 2017 was significantly higher than that of the area outside the working face. However, this vegetation coverage has decreased year by year since the mining in 2018 and reached a trough in 2019. It decreased more obviously for the working face underground mined in 2019, and was lower than the vegetation coverage on the right side for the first time. Then each area began to recover to the original distribution state before mining which showed a certain self-healing trend in 2020. In 2021, the distribution law of *FVC* inside and outside the working face returned to the similar state as in 2017, but compared with the area outside the mining face, the advantage of high vegetation coverage within the mining face has reduced.

**Fig 10:**
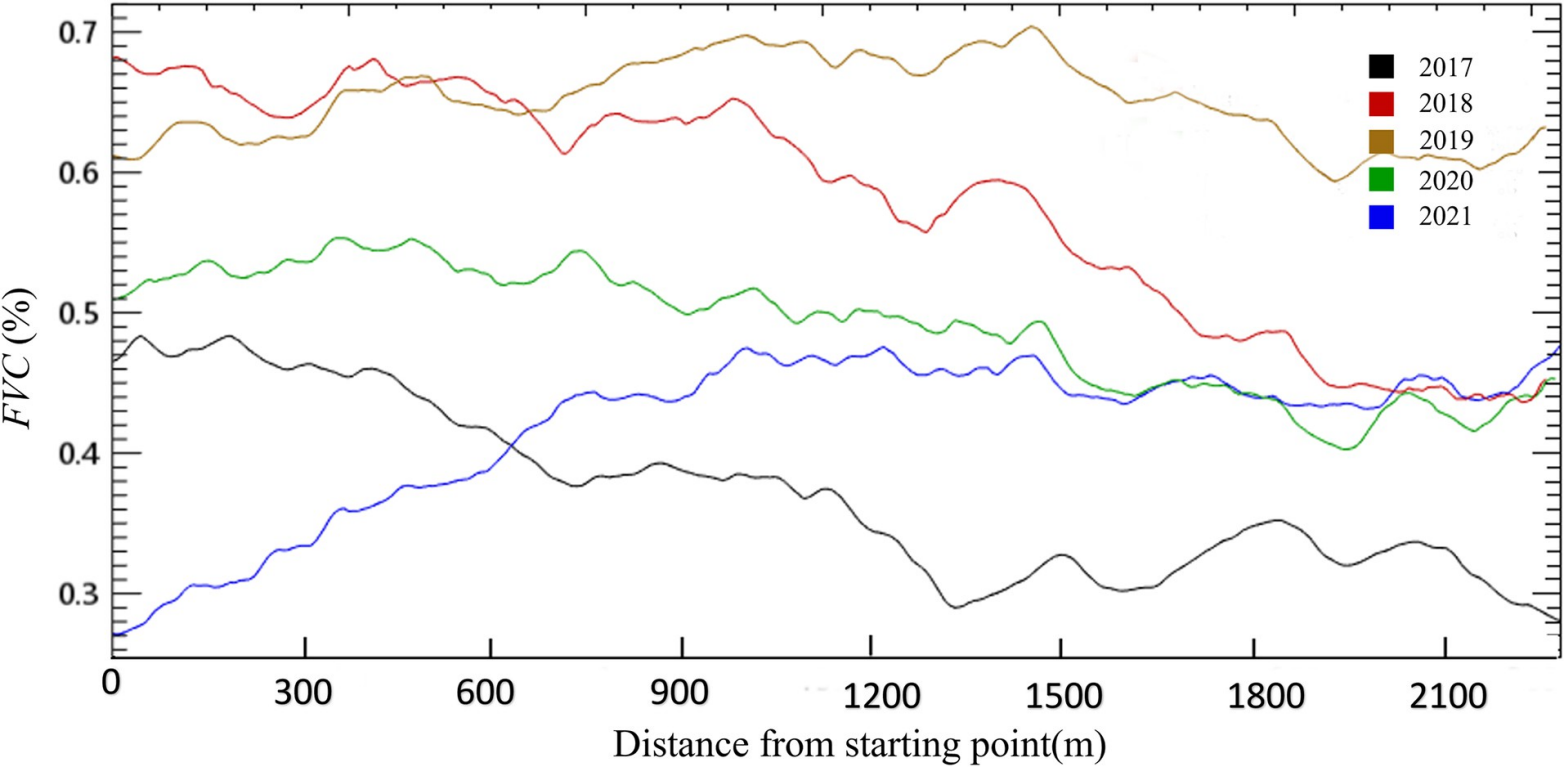
*FVC* change of the northern observation line.

The change trend of *FVC* was obtained for the northern observation line that crossed through two working faces underground mined in 2018 and 2019 as shown in Fig 10. It could be observed *FVC* distribution trend was high on the left and low on the right in the initial year 2017. Then it showed a certain disturbance effect and self-healing effect in subsequent years, but this effect was less obvious than that of the southern observation line. We could see the *FVC* increased in 2017–2018, and the working face underground mined in 2018 was not significantly disturbed. In 2019, the *FVC* of the area underground mined in 2018 was lower than that of the area underground mined in 2019. In 2020, the *FVC* in all regions decreased, but the decline in the area mined in 2019 was higher than that of the area mined in 2018. Besides, the *FVC* in the left area showed a larger decline, while the mining area was relatively stable in 2020–2021.

## Discussion

Underground mining has less direct destruction on the surface compared with open-pit mining. The impact of underground mining on surface vegetation includes the mining subsidence disturbance and change in groundwater level, which may have a certain impact on the growth of surface vegetation. Moreover, vegetation has a certain ability to resist stress, and can gradually adjust the growth to environmental changes [21]. In order to study and prove the above laws, some studies have studied the impact of coal mining subsidence or ground fissures on vegetation through surface sampling [22, 23]. However, long-term detection has rarely been carried out, and the vegetation change patterns of the entire surface working face before and after mining cannot be explained. In addition, some studies have used remote sensing satellite images to analyze vegetation coverage in mining areas [24, 25], but have not been able to compare and analyze different disturbed areas based on the location of the mining face. The main contribution of this study is to monitor and analyze the impact of different mining disturbance on vegetation coverage and post mining vegetation self-healing in the study area with multiple disturbance factors through Gaofen satellite remote sensing images. Considering the results of this study, the mining impact and self-healing laws were found and discussed as follows:

### Spatiotemporal variation of surface vegetation coverage affected by mining disturbances

In addition to the direct damage to the surface of the open-pit mining, its surrounding facilities and human activities will have a significant impact on the environment around the open-pit mine. Besides, precipitation is an important factor affecting vegetation growth [17]. The change of vegetation coverage in the entire study area was affected by climatic factors, and the number of precipitation days in each analysis area was moderately or strongly correlated with vegetation coverage. In addition, coal mining subsidence also has disturbance effects on surface vegetation. Generally speaking, the effects are difficult to detect only comparing the *FVC* on working face and control areas in the mining year, since there exist the vegetation coverage differences in the initial state due to the geomorphological factors. As in this study, when the area was not yet mined in 2017, vegetation coverage was not different in each analysis area. After the mining of the working face in 2018 and 2019, the vegetation coverage in the corresponding area on the surface showed a downward trend, but compared with the Natural growth control area in the same period, it was still at a high level although its growth rate decreased significantly. For the Open-pit mining affected area on the north side, the vegetation coverage was in a state of continuous decline, which is consistent with the disturbance law of the open-pit mining to the vegetation in other studies [26, 27].

It is noted that although the GaoFen multispectral images was used to explore the spatiotemporal changes of *FVC* on the working face scale level before and after mining, the temporal resolution of GaoFen data was limited. To reduce the impacts of phenology on vegetation, this study selected the peak month of vegetation growth for the research. And more details might be found if the data of different months could be obtained [28, 29]. Thus, the Unmanned Aerial Vehicle could be considered to obtain multispectral image of different months in the future since it was with the advantages of high temporal resolution, and less affected by weather and clouds.

### Differences in subsidence disturbance sensitivity of different landforms and surface vegetation and post-mining self-healing

It can be seen that different landforms and vegetation had different subsidence disturbance sensitivity according to the *FVC* change laws of the two observation lines laid out on different landforms. It could be attributed to the following three reasons:

1. Differences in landforms which leads to different mining damage or disturbance degree. The landform on the northern observation line was relatively flat, and the mining subsidence had less negative impact on the surface vegetation coverage. Generally, small fissures on the surface will disappear naturally within a few months after mining [30]. The landform of the southern observation line was relatively complex with large areas of gully and other fragmented landforms. The mining subsidence always had a chain reaction causing surface slope slippage, large-scale collapse or step fissures, etc., which would greatly disturb the surface vegetation [31, 32]. Previous studies on field testing have reported that ground fissures can cause damage to vegetation roots, soil moisture loss, and thus have a negative impact on vegetation growth [23, 33]. In this study area, the terrain on the south side is fragmented, and the development of ground fissures after mining is more severe than that in the flat area on the north side. This may also be one of the reasons for the relatively severe damage to vegetation coverage on the south side.
2. Differences in vegetation types. Plant roots and shoots are organic systems in a state of balance between supply and demand. Root damage will directly affect the root system’s ability to support nutrients, once the nutrient requirements of shoots cannot be met, it will seriously affect plant growth [34]. It could be observed that the northern observation line was mostly covered with herbaceous shrubs, while the southern observation line had a large number of woody plants according to remote sensing image and field investigation. Some studies have reported that the root system of small vegetation on the surface of the mining area had a certain self-healing effect after mining disturbance [35]. Compared with herbaceous shrubs, the root system of woody plants is huger with greater horizontal range and depth and is more susceptible to severe damage in the subsidence disturbance. Besides, the huge aboveground nutrient demand of woody plants will further increase the insufficient nutrient supply pressure of damaged roots.
3. Differences in soil moisture. It could be seen that there was a strong correlation between the two working face areas and the number of precipitation days (the correlation coefficient was higher than that of the natura growth control area), which indicated that the importance of precipitation in the subsidence area was more prominent. Soil water storage capacity was different in different regions, and the fractured landscape was affected by soil landslides and large cracks which may lead to the decrease of the soil water retention capacity. Therefore, the supply of soil moisture may be an important factor leading to different degrees of surface vegetation recovery after mining.

### Post-mining self-healing effect and anti-disturbance characteristics of vegetation in reclaimed Old mining areas

As to the observation lines, it could be seen that the vegetation coverage of the working face after mining decreased significantly, but with the passage of time, it gradually approached the level in 2017. This reflected that the surface vegetation had a self-healing effect after mining. The surface vegetation would gradually adapt to the mining stress, thereby reduce the ecological disturbance effect of the mining stress over time. According to the recovery trend of *FVC*, it can be roughly inferred that the recovery to the state before mining is about 3 years after mining. Besides, the correlation coefficient *r* between the Old mining area and the number of precipitation days is significantly higher than that of natural growth control area, and in years with less precipitation, the growth rate of vegetation coverage was lower than that in the control area. Field investigations showed that although reclamation had been carried out in the Old mining area, the types of vegetation planted were relatively simple compared to natural growth control area. It was reported that the vegetation type used for land reclamation in mining areas had an impact on the functional genes and enzyme activities of soil microorganisms [36], which may lead to a decrease in its ability to resist stress and adapt to the natural environment.

## Conclusion

To explore the impact of different mining disturbance on vegetation coverage and post mining self-healing of vegetation, this paper used GaoFen multispectral images and divided the study area into the Underground mining area 2018, Underground mining area 2019, Old mining area, Open-pit mining affected area and Natural growth control area. The spatiotemporal changes of *FVC* were analyzed in each area and the correlation between vegetation coverage and climatic factors was studied. The main conclusions were as follows:

1. The overall vegetation coverage in the study area showed a slight decline trend, with the most significant decline in the Open-pit mining affected area. In other areas the *FVC* fluctuated in different years. Each analysis area was significantly affected by the natural climate, and each analysis area was positively correlated with the number of precipitation days.
2. Underground mining in the study area had a negative impact on *FVC*. Working faces in 2018 and 2019 showed a restraint on the growth of surface vegetation. Topographical factors might cause differences in sensitivity of vegetation coverage changes on the surface, showing that the southern fractured landform was more sensitive to mining disturbances, while the northern flat landform was less sensitive to mining disturbances. Although the surface vegetation coverage of both working surfaces was affected after mining, it showed certain self-healing phenomena over time.
3. Vegetation types in the reclaimed Old mining areas were relatively single compared with Natural growth control areas. Although *FVC* in Old mining areas was higher, but its resistance and stability were weak.

The findings of this study provided guidance for post-mining ecological restoration and land reclamation. It is recommended that different vegetation restoration strategies be implemented based on the specific disturbance levels in different areas. Furthermore, natural factors such as terrain and climate should be effectively utilized to enhance the effectiveness of the restoration efforts. When conducting land reclamation, it is crucial to consider the appropriate selection of native plant species to avoid the poor environmental resilience caused by a lack of vegetation diversity. Additionally, the monitoring methods used in this study can contribute to post-mining ecological monitoring. For future research, higher-resolution satellite or unmanned aerial vehicle image can be utilized to investigate the variations in vegetation types and diversity across different post-mining regions. Correlation analyses can also be conducted to examine the spatial relationships between soil nutrient variations and their impact on vegetation growth, which might further reveal the disturbance mechanisms of mining activities on vegetation.
